# Mitochondrial Dysfunction, Protein Misfolding and Neuroinflammation in Parkinson’s Disease: Roads to Biomarker Discovery

**DOI:** 10.3390/biom11101508

**Published:** 2021-10-13

**Authors:** Anna Picca, Flora Guerra, Riccardo Calvani, Roberta Romano, Hélio José Coelho-Júnior, Cecilia Bucci, Emanuele Marzetti

**Affiliations:** 1 Fondazione Policlinico Universitario “Agostino Gemelli” IRCCS, 00168 Rome, Italy; anna.picca@policlinicogemelli.it (A.P.); emanuele.marzetti@policlinicogemelli.it (E.M.); 2 Aging Research Center, Department of Neurobiology, Care Sciences and Society, Karolinska Institutet and Stockholm University, 17165 Stockholm, Sweden; 3 Department of Biological and Environmental Sciences and Technologies, Università del Salento, 73100 Lecce, Italy; flora.guerra@unisalento.it (F.G.); roberta.romano@unisalento.it (R.R.); cecilia.bucci@unisalento.it (C.B.); 4 Department of Geriatrics and Orthopedics, Università Cattolica del Sacro Cuore, 00168 Rome, Italy; coelhojunior@hotmail.com.br

**Keywords:** alpha-synuclein, beta-amyloid (Aβ), cytokine, DAMPs, extracellular vesicles, inflammation, mitochondrial-derived vesicles, mitophagy, neurofilaments light chain (NfL), p-tau pathology

## Abstract

Parkinson’s Disease (PD) is a highly prevalent neurodegenerative disease among older adults. PD neuropathology is marked by the progressive loss of the dopaminergic neurons of the substantia nigra pars compacta and the widespread accumulation of misfolded intracellular α-synuclein (α-syn). Genetic mutations and post-translational modifications, such as α-syn phosphorylation, have been identified among the multiple factors supporting α-syn accrual during PD. A decline in the clearance capacity of the ubiquitin-proteasome and the autophagy-lysosomal systems, together with mitochondrial dysfunction, have been indicated as major pathophysiological mechanisms of PD neurodegeneration. The accrual of misfolded α-syn aggregates into soluble oligomers, and the generation of insoluble fibrils composing the core of intraneuronal Lewy bodies and Lewy neurites observed during PD neurodegeneration, are ignited by the overproduction of reactive oxygen species (ROS). The ROS activate the α-syn aggregation cascade and, together with the Lewy bodies, promote neurodegeneration. However, the molecular pathways underlying the dynamic evolution of PD remain undeciphered. These gaps in knowledge, together with the clinical heterogeneity of PD, have hampered the identification of the biomarkers that may be used to assist in diagnosis, treatment monitoring, and prognostication. Herein, we illustrate the main pathways involved in PD pathogenesis and discuss their possible exploitation for biomarker discovery.

## 1. Introduction

Parkinson’s Disease (PD) is a highly prevalent neurodegenerative disease among older adults, which is likely to be due to the worldwide demographic transition, longer disease duration, and higher exposure to environmental risk factors [1,2].

**Figure 1:**
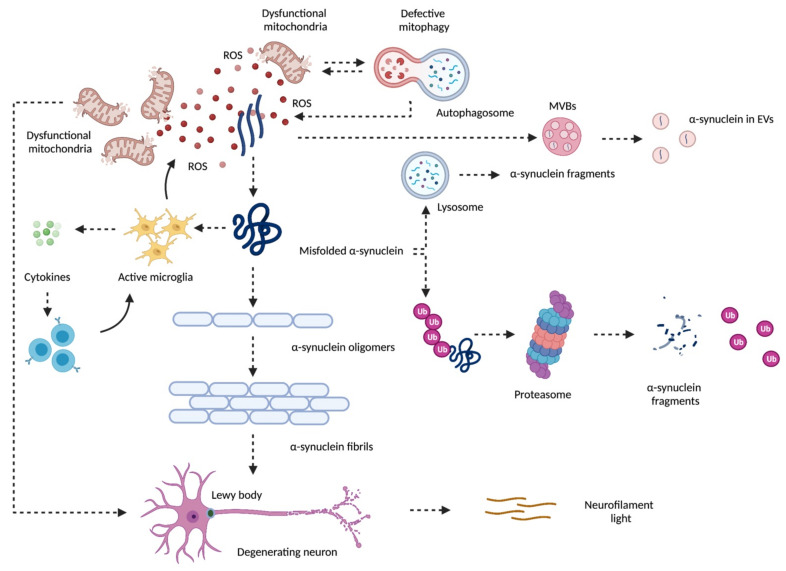
Schematic representation of the contribution of defective mitochondrial quality control and mitochondrial dysfunction to neurodegeneration in Parkinson’s disease. Mitochondrial dysfunction arising from defective quality control pathways (e.g., mitophagy) ignites the production of reactive oxygen species (ROS) and activates the α-synuclein aggregation cascade. Before reaching its pathological conformation, α-synuclein can be degraded along the endosomal pathway, packaged within multivesicular bodies (MVBs), and released as extracellular vesicles (EVs). In the setting of overwhelmed ubiquitin-proteasome and autophagy-lysosomal systems, misfolded α-synuclein is not efficiently cleared and aggregates into soluble oligomers. These structures generate insoluble fibrils that compose the core of intraneuronal Lewy bodies and Lewy neurites, thereby promoting neurodegeneration. The accrual of misfolded α-synuclein activates microglial cells that attract cells of peripheral immunity within the central nervous system via cytokine release. This instigates a vicious circle resulting in persistent microglial activation which amplifies mitochondrial dysfunction and induces further neuronal damage. Created with BioRender.com, accessed on 9 October 2021.

PD neuropathology is marked by the progressive denervation of the dopaminergic neurons of the substantia nigra pars compacta and the widespread accumulation of misfolded intracellular α-synuclein (α-syn) [3]. Several factors, including genetic mutations and post-translational modifications, such as α-syn phosphorylation, support α-syn accrual during PD. Indeed, in physiological conditions misfolded α-syn can be cleared via the ubiquitin-proteasome and the autophagy-lysosomal systems. However, an increase in misfolded proteins that overwhelms the cell’s clearance capacity by engulfing the ubiquitin-proteasome and the autophagy-lysosomal systems is a main pathophysiological mechanism of PD neurodegeneration (reviewed in [4]). In this setting, the misfolded α-syn aggregates into soluble oligomers and generates insoluble fibrils which compose the core of the intraneuronal Lewy bodies and Lewy neurites. Along with this, mitochondrial dysfunction triggered by a genetically driven loss of function of the mitophagy factors phosphatase and tensin homolog-induced kinase 1 (PINK1), deglycase DJ-1, or leucine-rich repeat kinase 2 (LRRK2) can ignite the production of reactive oxygen species (ROS) and activate the α-syn aggregation cascade that, together with the Lewy bodies, promotes neurodegeneration (reviewed in [4]). Once formed, aggregates of the misfolded α-syn instigate inflammation-mediated activation of microglial cells that attract the cells of the peripheral immunity within the central nervous system (CNS) via the release of inflammatory mediators (reviewed in [4]). Here, the activation of resident lymphocytes leads to persistent microglial activation that amplifies mitochondrial dysfunction and causes further neuronal damage (Figure 1).

Although great advances have been made towards the identification of the cellular and molecular mechanisms involved in PD neurodegeneration, several aspects of its pathophysiology and, more specifically, those featuring the dynamic evolution of the disease remain undeciphered. These gaps in knowledge, together with the clinical heterogeneity of PD, have hampered the identification of biomarkers that may be used to aid in the diagnosis, treatment monitoring, and prognostication [5].

A wide range of clinical, biochemical, imaging, and genetic parameters have been investigated as possible biomarkers of PD [5,6]. Most studies were based on single marker approaches [7]. More recently, innovative multi-marker strategies have been applied to allow a more comprehensive appraisal of the complex conditions, including PD [8,9,10,11,12]. These strategies combine high-throughput analytical platforms to measure panels of circulating mediators related to different biological domains and multivariate statistical analysis [8,9,10,11,12]. Through this approach, amino acid signatures [13] and extracellular vesicles-associated mitochondrial fingerprints [14] have been identified in the serum of older adults with PD in the EXosomes in PArkiNson Disease (EXPAND) study [15]. In the attempt to evaluate the contribution of neuroinflammation and amino acid metabolism to PD, these findings have been complemented with markers of systemic inflammation, neurogenesis, and neural plasticity in a subsequent investigation in which specific patterns of these mediators were identified as robust predictors of PD [12].

However, additional disease-specific biomarkers that could mirror the multifaceted characteristics of PD pathogenesis at the systemic level are highly sought after. To this end, ad hoc translational studies which include both pre-clinical and clinical information and focus on major PD-related mechanisms are needed. In particular, a deeper investigation of the signaling pathways involving α-syn, neuroinflammation, beta-amyloid (Aβ) and p-tau pathology, neurofilaments light chain (NfL), and axonal injury and degeneration may provide unprecedented information on PD pathophysiology and contribute substantially to the biomarker discovery process. A combination of α-syn species, lysosomal enzymes, markers of amyloid and tau pathology, and NfL in the cerebrovascular fluid (CSF) and blood have been reported as potential diagnostic and prognostic biomarkers of PD, reflecting the dynamic course of its pathophysiology [4] (Table 1). However, further validation of these mediators in large independent cohorts is required for their adoption in the clinical setting to improve PD diagnostic and prognostic accuracy. 

Herein, we illustrate the main pathways involved in PD pathogenesis and discuss their possible exploitation for biomarker discovery.

## 2. Mitochondrial Dysfunction and Neurodegeneration in Parkinson’s Disease

Mitochondrial dysfunction is a well-recognized initiating factor of dopaminergic neuronal degeneration and mainly results from the inhibiting effect of aberrant α-syn and toxins at the level of complex I of the electron transport chain [28]. Indeed, dysfunctional complex I has been identified in the brain samples of people with sporadic PD, while impaired mitophagy and altered mitochondrial quality control have been reported in the familial forms of early-onset PD due to mutations in the autosomal recessive genes Pink1 and Parkin [29].

The role of the PINK1/Parkin pathway in the maintenance of mitochondrial quality control has been thoroughly investigated [30] and its implications in PD pathophysiology and therapeutic developments have also emerged [31]. According to the latest model of the PINK1-mediated activation of Parkin, a loss in mitochondrial membrane potential triggers the stabilization of PINK1 at the outer mitochondrial membrane (OMM), whereby it phosphorylates a pre-existing ubiquitin (Ub) residue at the serine 65 (Phospho-serine65 Ub; pSer65Ub) [32]. This event signals the recruitment of auto-inhibited cytosolic Parkin to the sites of PINK1 and enables the binding of the ring finger protein 1 of the Parkin domain to the pSer65Ub, thus initiating a series of conformational changes at the level of the OMM [33]. While bound to pSer65Ub, Parkin remains quiescent and becomes fully activated following the phosphorylation of its Ub-like domain by PINK1 [34]. Because of this key role in the activation of PINK1, pSer65Ub is increasingly appreciated as a marker of active PINK1-induced mitophagy that ultimately leads to the recruitment of Parkin in a feedforward mechanism. Indeed, once active, Parkin initiates a cascade of Ub chain synthesis on the OMM proteins that provide substrates for further PINK1 ubiquitin phosphorylation that, in turn, recruits more Parkin. This cascade of events culminates into a massive activation of the Ub chain assembly [35]. An increase in pSer65Ub levels has been observed in the postmortem brains of people with PD, while lower pSer65Ub has been identified in the familial forms of PD holding Pink1/Parkin mutations, thereby indicating the relevance of this pathway during PD neurodegeneration [36,37].

In addition to this, the mutation of the genes Snca and the leucine-rich repeat kinase 2 (Lrrk2) and their implication in PD-related mitochondrial dysfunction are well-established. LRRK2 is a widely expressed multidomain kinase that has been shown to regulate mitophagy in iPSC-derived dopamine neurons by removing the OMM adaptor protein MIRO from the MIRO/MILTON/KINESIN motor complex. This action reduces the mitochondrial transport along the cytoskeleton and alleviates the autophagosome engulfment throughout the cell, thus favoring organelle clearance [38]. The G2019S mutation in the kinase domain of LRRK2 disrupts this function and slows down mitophagy initiation [38]. Impaired mitochondrial trafficking has also been observed in rat neurons carrying Lrrk2-R1441C mutations [39] and neuronal cultures from patients with idiopathic PD, thus supporting the role of compromised mitophagy in the pathology of PD [38]. Mutant LRRK2 can also impair PINK1/Parkin-dependent mitophagy via other mechanisms [40,41]. In particular, an increase in the kinase activity of LRRK2-G2019S mutants is able to disrupt the protein–protein interactions at the OMM early in the PINK1/Parkin-dependent mitophagy pathway [40]. Furthermore, Lrrk2-G2019S and R1441C mutations have also been implicated in the impairment of the later stages of the PINK1/Parkin-dependent mitophagy via the increasing of the phosphorylation of RAB10, a small GTPase regulating intracellular membrane trafficking. Indeed, this event inhibits the interaction of RAB10 with the autophagy receptor optineurin and reduces the accumulation of PINK1/Parkin on the depolarized mitochondria [41]. 

Finally, mutations in the Chchd2 gene encoding for a protein-regulating mitochondrial function have been identified as a novel risk gene for familial PD. CHCHD2 protein is involved in preserving mitochondrial cristae [42] and promoting mitochondrial fusion by stabilizing optic atrophy 1 (OPA1) [43], and it accumulates in damaged mitochondria. When mutated, CHCHD2 induces the precipitation of intermembrane space proteins [43] and the destabilization of cytochrome C, which ultimately results in impaired mitochondrial bioenergetics and high mitochondrial ROS generation [44]. Mutations in CHCHD2 have also been associated with the aggregation and oligomerization of α-synuclein in the postmortem brain tissues of people with PD and iPSC-derived dopaminergic neurons [45]. Although LRRK2 and CHCHD2 mutations have been implicated in mitochondrial homeostasis, their role as initiators of mitochondrial dysfunction via aberrant mitophagy warrants investigation.

## 3. α-Synuclein

Clinical signs of PD appear when 70–80% of the dopaminergic terminals in the striatum and about 50% of the dopaminergic neurons in the substantia nigra have been lost. This long presymptomatic phase of the disease makes it particularly difficult to tease out the molecular events responsible for the initiation and progression of neurodegeneration [46]. Biomarkers associated with early PD are highly sought after as they may help to identity molecular targets and design therapeutic strategies to prevent the degeneration of dopaminergic neurons. In addition to this, the treatment of PD with drugs able to modify the progression of the disease is hampered by the lack of sensitive and specific biomarkers. Indeed, the observation of motor symptoms at the clinical level is still the sole criterion for the diagnosis of PD, with the inevitable downsides of incurring mistakes in the diagnosis, particularly in early disease stages [47]. 

The biomarker discovery process for PD has gained increasing attention, with a special interest in the identification of the proteins/nucleic acids associated with extracellular vesicles (EVs) isolated from biofluids (i.e., plasma/CSF) [48,49]. EVs are, indeed, promising biomarker candidates for PD as they carry a plethora of biomolecules and convey information on the originating cells [50]. First, the core of the intracellular pathogenic processes is reflected by EV cargos holding cell-type-specific proteins and nucleic acids. Second, EVs can cross the blood-brain barrier (BBB). Therefore, EVs originating in the brain reach the peripheral blood from which they can be isolated and characterized for the identification of biomarkers of CNS disease.

As discussed earlier, the presence of Lewy bodies is one of the major pathological traits of PD [51]. The main component of Lewy bodies and Lewy neurites, both in the genetic and the sporadic forms of PD, is the misfolded α-syn in fibrils [51]. In addition, ROS bursts, neuroinflammation, excitotoxicity, apoptosis, and the loss of trophic factors act synergistically to induce the degeneration of dopaminergic neurons [52]. Spatio-temporal aggregation of α-syn and its contribution to neurodegeneration have been characterized in mouse models of PD obtained by intracerebral injection of α-syn pre-formed fibrils (α-syn-PFF) [53,54]. These mice gradually develop inclusions of aggregated α-syn and α-syn-mediated neuronal loss [55] and offer relevant information on the processes (e.g., basal ganglia synaptic plasticity and the mechanisms underlying motor-learning processes) that are altered before massive neurodegeneration occurs [56,57,58,59]. The α-syn-PFF mouse model allows detailed evaluation of the early and progressive neuronal and synaptic dysfunctions that precede complete dopaminergic denervation and that can be correlated with motor/behavioral impairments [54,58].

One of the strategies to prevent the progression of PD involves targeting the formation and the neuron-to-neuron spread of abnormal α-syn aggregates. The first line of evidence supporting the release of pathogenic α-syn was the demonstration of aggregates in the CSF [60]. The α-syn shuttles between host neurons and the neighboring cells [61,62], and the uptake of α-syn oligomers is facilitated by their incorporation into EVs [61]. Indeed, EVs are also involved in the regulation of the intercellular communication between neuronal cells to modulate the expression of local proteins and trigger localized signaling events and synapse formation [63]. Moreover, neurons can use EVs to remove unwanted materials. Hence, EV-associated α-syn could facilitate two separate processes: (i) the diffusion of pathogenetic insults by cell-to-cell transfer mechanisms; (ii) the elimination of toxic α-syn species to maintain neuronal homeostasis. The selection between these two scenarios seems to depend upon the mechanism of the EV α-syn uptake and the recipient cell type. The transmission of aberrant α-syn species through synaptically connected neurons requires the endocytosis of pathological α-syn, which is facilitated by its association with EVs [64,65]. On the other hand, microglia and astrocytes, which are responsible for maintaining microenvironment homeostasis in the brain, internalize EVs to mediate the removal of the EV-associated α-syn aggregates [66]. In this regard, microglia are more efficient in taking up the EV-associated extracellular α-syn compared with the neurons or astrocytes [61]. After internalization, the EV-associated α-syn is directed to the lysosomes for degradation. When astrocytes take up the EV-associated α-syn, they can induce the production of pro-inflammatory cytokines and chemokines [67]. Moreover, the astrocytes are able to both secrete and receive EVs from other cell sources [68]. Ngolab et al. [69] have recently reported that the injection of human brain-derived EVs carrying pathological forms of α-syn in mouse brains induce α-syn aggregation in astrocytes to a greater extent than in the neurons. The aforementioned mechanisms cooperate to clear extracellular α-syn, and alterations in any of these pathways potentially determine a destabilization of the system leading to further aggregation and propagation of α-syn. 

The aggregation of α-syn induces neuronal death [70]. The EVs containing α-syn have been detected in the separated conditioned media of SH-SY5Y cells with wild-type α-syn and inducible β-galactosidase, and EV-associated α-syn impacts on the viability of neighboring neurons [71]. In particular, the overexpression of α-syn in SH-SY5Y cells induces an efficient transfer of α-syn by an EV route to normal SH-SY5Y cells and, when the secretion of EVs increases, its transmission to recipient cells increases accordingly [72]. The EV lipid composition can also accelerate the aggregation of exogenous α-syn [73]. Chang et al. [74] observed that an increase in EV secretion by microglial BV-2 cells was induced by α-syn following an increase in the apoptosis of cortical neurons. These observations allowed the authors to conclude that α-syn-induced neurodegeneration in PD was mediated by EV-activated microglia. Kunadt et al. [75] provided evidence for EV-associated α-syn in the CNS in vivo. The authors observed that EVs from CFS contain a pathogenic species of α-syn that was able to initiate the oligomerization of soluble α-syn in target cells in a dose-dependent manner and to confer disease pathology [75]. These analyses support the hypothesis that EVs contain “seed” and “strains” to determine the spreading of α-syn aggregates in the brain and support the pathogenetic pathways.

Neurons can also benefit from the reduction in the intracellular levels of α-syn via their secretion into EVs, thereby acting as friends or foes in neurodegeneration [48]. Surviving dopaminergic neurons of the substantia nigra of people with PD hold higher levels of ATP13A2 mRNA and protein than the controls [76,77]. Gitler et al. [78] showed that ATP13A2 had an inhibitory effect on the α-syn toxicity in primary dopaminergic neurons. Indeed, a greater expression of ATP13A2 may increase EV-associated α-syn secretion with the consequent reduction in intracellular α-syn levels [79]. In contrast, a reduced expression of ATP13A2 leads to decreased levels of EV-associated α-syn [79]. According to these findings, indicating a potential neuroprotective role of EVs in PD, α-syn was reported to be significantly lower in the CSF of people with PD relative to controls [80,81,82]. Moreover, a decrease in EV-associated α-syn levels was found in the CSF of people with PD, consistent with total α-syn levels in the CSF [83]. However, high levels of EV-associated α-syn were observed in the peripheral blood. The use of the anti-L1CAM (neural cell adhesion molecule L1) antibody, which specifically identifies circulating EVs derived from the CNS, allowed the identification that CNS-derived EVs can efflux into the blood. Moreover, the levels of α-syn in CNS-derived EVs isolated in plasma are substantially higher in people with PD and are associated with the severity of the disease [84]. Therefore, plasma CNS-derived EV α-syn can serve as a novel PD biomarker with high sensitivity and specificity [84]. 

## 4. β-Amyloid and p-Tau Pathology

PD and Alzheimer’s disease (AD) are the two most common neurodegenerative disorders and share some pathological traits, such as misfolding, aggregation, and widespread accumulation of natively unfolded proteins [85,86,87]. Amyloid beta (Aβ) deposits into extracellular Aβ plaques and the hyperphosphorylation of Tau protein, leading to the formation of neurofibrillary tangles (NFT) inside neurons, are specific alterations of AD [88].

Cognitive impairment occurs in most people with PD during the natural course of the disease [89]. Interestingly, beyond α-syn pathology [90,91], diffuse Aβ plaques (a feature of AD) [92] have been found in the striatum of PD-associated dementia (PDD) compared with PD without dementia and may represent a possible substratum for cognitive impairment. Indeed, despite the central role of α-syn pathology in PDD, several studies also indicate that the cognitive status of PDD correlates with the deposits of Aβ plaques and Tau NFT [93,94,95,96,97]. AD-like neuropathology seems to be a more specific correlate of dementia in PD than α-syn pathology [93,98]. Indeed, a recent study identified that the combination of the measures of cortical α- syn, Tau, and Aβ pathologies was the best predictor of dementia in PD compared to any single marker alone [93].

Tau is encoded by the microtubule association protein Tau (*MAPT)* gene, which in humans is located on chromosome 17. Hereditary dominant frontotemporal dementia with parkinsonism in chromosome 17 (FTDP-17) is linked to *MAPT* mutations and includes diverse clinical syndromes and various anatomical distributions of Tau inclusions depending on the specific mutations [99]. Of note, studies by Polanco et al. [100,101,102] have shown that exosomes, secretory nanovesicles generated by late endosomes, are relevant mediators of the trans-synaptic spreading of Tau seeds and a route in the pathogenic mechanisms guiding the progression of tauopathies. The authors showed that brain-derived exosomes isolated from Tau transgenic rTg4510 mice contain Tau seeds that are able to induce Tau protein aggregation in recipient cells [100]. This role of exosomes in spreading Tau seeds through interconnected neurons is achieved by hijacking the endosomal pathway [101]. In a subsequent study, the mechanisms whereby exosomes-internalized Tau seeds exploit mechanisms of lysosomal degradation to elude endosomes and trigger cytosolic Tau aggregation have been characterized in HEK293T-derived ‘Tau biosensor cells’ [102]. The authors found that the majority of endosomes containing exosomes were fused with lysosomes to form endolysosomes. Herein, the exosomes were able to permeabilize these endolysosomes, irrespective of the presence of the Tau seeds, both in the non-neuronal Tau biosensor cells and the primary neurons with a threshold-guided mechanism [102]. However, the aggregation of Tau was triggered only in cells that showed permeabilization, thereby lending an escape route to the exosome-associated Tau seeds into the cytoplasm [102]. In the same study, it was also shown that the overexpression of the GTPase Ras-associated binding 7 (RAB7) protein, which is required for the fusion of endosome with lysosomes, strongly enhanced Tau aggregation. This event was, instead, inhibited by knocking down RAB7 or by using alkalinizing agents that block lysosomal function. Therefore, the authors concluded that the activity of lysosomal enzymes permeabilizes exosomal and endosomal membranes and grants access to exosomal Tau seeds into the cytoplasm. Here, Tau seeds that were able to resist the acidic endolysosomal environment and the activity of proteases induced auto-aggregation, thus also underscoring the relevance of endosomal membrane integrity in the process of cellular invasion by misfolded proteins [102].

Some evidence suggests that classic AD biomarkers in CSF may be predictive of future cognitive impairment in people with PD. Several studies observed that lower baseline concentrations of Aβ42 in the CSF were associated with worse cognition and predicted cognitive decline in people with PD, and transition to PDD [21,103,104,105], while CSF total Tau and phosphorylated Tau showed inconsistent findings [104,106,107,108]. In addition to this, the evaluation of Aβ42 in the CSF together with other CSF biomarkers, in combination with the clinical features, improve the prognostic value of this biomarker. Notably, low CSF Aβ42 levels have been found to predict early psychosis in people with PD and are associated with the appearance of illusions or hallucinations within 3–4 years of follow-up [109].

Different from Aβ42 in the CSF, the determination of a-syn as a predictor of cognitive impairment has shown conflicting results. The worsening of cognitive function and, in particular, verbal memory and information processing speed over time was associated with higher CSF total α-syn [103,110]. However, low concentrations of total α-syn in the CSF have also been identified as a predictor of cognitive decline [111]. In other studies, no prognostic information of CSF total α-syn and oligomeric α-syn have been determined [21,112,113,114]. In a recent work, Parnetti et al. [115] performed a prospective study in 44 PD patients and 25 controls with other neurological conditions to verify whether the combination of CSF α-syn species and classical AD biomarkers may predict PD and cognitive decline. The authors confirmed that low levels of Aβ42 in the CSF were an α-syn-independent predictive factor for cognitive decline in PD, while no prognostic value was determined for any of the other assessed biomarkers, (i.e., total/phospho Tau and total/oligomers α-syn) [115].

## 5. Neurofilament Light Chain and Axonal Injury during PD Neurodegeneration

Neurofilaments (Nfs) are multi-subunit type IV intermediate filaments that compose the scaffolding of the axonal cytoskeleton. These structural proteins are essential for preserving axonal caliber, stability, growth, and transmission of electrical impulse [116,117,118]. The protein subunits forming the Nf vary in molecular mass, a characteristic that allows their classification into heavy (NfH, 210 kDa), median (NfM, 150-190 KDa), and light Nf polypeptides (NfL, 68 kDa), and α-internexin (Int). The concentration of these different subunits within the axonal structures is uneven, while NfL is the most abundant and soluble protein form. 

The axoplasm of large myelinated axons are enriched in Nfs that have been found to be released from the axons in an age- and sex-dependent manner, thereby reflecting physiological aging [119,120,121]. However, in conditions promoting axonal damage, such as neurodegeneration, the release of Nfs into the extracellular space, the CSF, and eventually the blood, increases significantly and for this reason is proposed as a marker of white matter axonal degeneration [122]. Indeed, the release of Nfs has been reported to increase according to the extent of axonal damage in multiple sclerosis and their potential role as markers of disease activity has been widely acknowledged [123,124,125,126,127,128,129,130,131,132]. Furthermore, the variation of the circulating levels of Nfs following disease worsening or improvement indicates that Nf release is highly sensitive to disease activity and progression [125]. However, the abundance of different Nf isoforms into the circulation is also likely indicative of different neurodegenerative processes [133]. For instance, the NfH subunit, which undergoes extensive phosphorylation and plays a major role in the dynamics of axonal transportation, has been indicated as a more specific marker of amyotrophic lateral sclerosis [134]. NfL levels have been reported to increase during relapses of multiple sclerosis and to correlate with lesion development [125,135,136,137], disability, and disease activity and progression [138].

Besides the very informative role of this circulating mediator in different settings, the development of minimally invasive and cost-effective sensitive assays for the quantification of NfL in biofluids of different origin has boosted the evaluation of this mediator as a biomarker of neurodegeneration. Indeed, the implementation of novel analytical platforms also makes possible the quantification of NfL in blood, where the concentrations of NfL are about 40-fold lower than in the CSF. Furthermore, the simplicity and safety of NfL measurement has allowed its inclusion in clinical trials testing the effects of disease-modifying therapies [139,140,141,142,143]. These findings hold hope towards making NfL a valuable outcome measure in clinical trials of multiple sclerosis and other neurodegenerative conditions [140].

With regard to PD, no strong evidence exists on the differences in CSF and blood concentrations of NfL in people with PD compared with the controls. Indeed, white matter axonal degeneration is not typical of PD, at least in the disease’s early stages. Conversely, NfL levels have been shown to be higher in the CSF of people with atypical parkinsonism (i.e., progressive supranuclear palsy, multiple system atrophy, and corticobasal syndrome), in which a remarkable involvement of myelinated axons during the neurodegenerative process occurs [144]. In PD, the release of NfL may be relevant in the later stages of the disease and warrants investigation [4]. 

There are some relevant considerations that deserve discussion when planning to quantify and use NfL measurements in clinical practice for PD. NfL does not represent a biomarker specific to PD because other neurodegenerative diseases (i.e., multiple sclerosis, prion diseases, amyotrophic lateral sclerosis, AD, Huntington’s disease, and traumatic brain injury) share an increase in circulating levels of NfL [120,122]. Furthermore, the assessment of systemic NfL concentrations does not allow the making of inferences about the site of axonal damage [125]. More relevantly, the diagnostic accuracy of circulating NfL is challenged by the multiple findings indicating that NfL is also elevated in people with peripheral nerve disease [145,146]. Finally, changes of the circulating levels of NfL according with age, sex, and body mass index, and blood volume have been reported [147], and the lack of reference values within the general population makes difficult the evaluation of the optimal sampling frequency to be adopted in clinical studies. Longitudinal measurements are still the preferred strategy for achieving differential diagnosis between PD and atypical parkinsonian disorders and support clinical decision-making until normative values are established [130,148].

## 6. Neuroinflammation

Neuroinflammation is a hallmark of PD and a major ignitor of the degeneration of dopaminergic neurons [149,150,151,152]. While an inflammatory response is mounted under physiological conditions to preserve CNS homeostasis, a detrimental effect of inflammation may arise from its chronic activation. The control over inflammation within the CNS is performed by the microglial cells that, together with perivascular macrophages, act as resident immune sentinels within the brain parenchyma and cooperate with neuronal cells to ensure CNS health. When a breach in the CNS microenvironment occurs, microglial cells acquire either a pro- or an anti-inflammatory phenotype, depending on the CNS alterations to be buffered. In the setting of primary neurodegeneration, axonal degeneration, and/or peripheral inflammatory processes, and excessive activation of the proinflammatory microglia may trigger chronic inflammation. Under these circumstances, harmful processes may be elicited which ultimately favor neuronal loss [149,150,151,152].

One of the mechanisms linking neuroinflammation to PD pathophysiology is related to its role in the initiation of α-syn aggregation and propagation and microglial cell activation [153]. Indeed, the substantia nigra pars compacta is highly dense in microglia [154,155] and is characterized by the presence of dopaminergic neurons that are highly and selectively vulnerable to inflammatory attacks [155,156,157,158]. Indeed, high levels of α-syn-specific T cell responses may occur years before the manifestation and diagnosis of PD, thereby indicating a role for neuroinflammation in PD pathogenesis and a possible target for early diagnosis and treatment [153]. Results from studies in rats overexpressing α-syn support the notion that alleviating neuroinflammation may ameliorate symptoms in early PD [159]. In particular, targeting the inflammatory response in α-syn overexpressing rats by administering interferon γ and resolvin D1 has been shown to prevent central and peripheral inflammation, as well as neuronal dysfunction and motor deficits [159].

**Figure 2:**
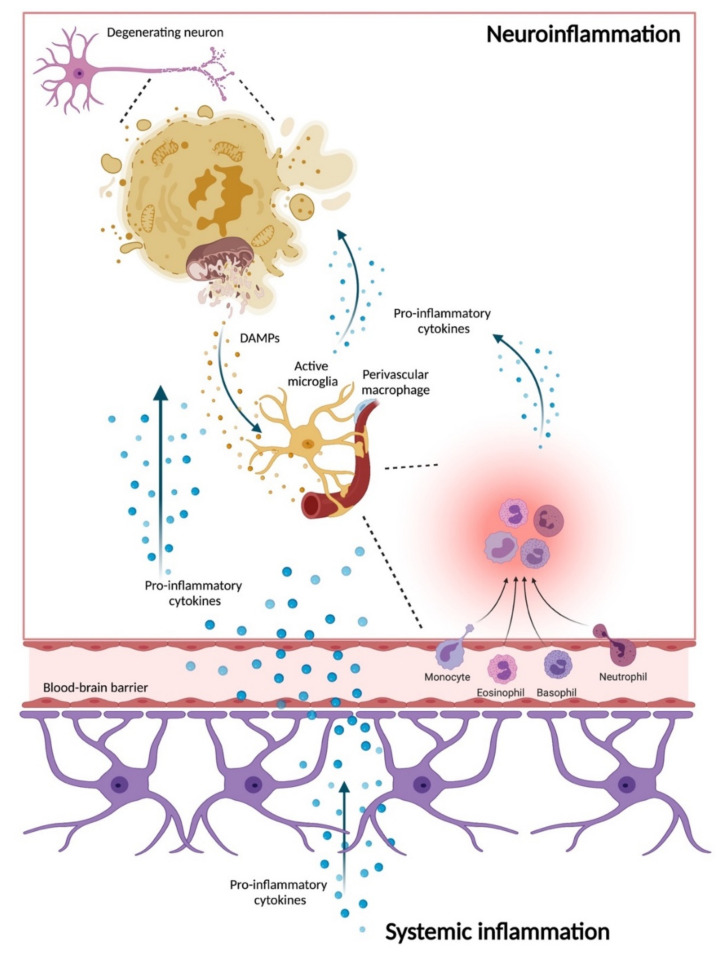
Schematic representation of the events linking peripheral and neuronal inflammation in Parkinson’s disease. The release of damage-associated molecular patterns (DAMPs) from damaged neurons, including mitochondrial DNA, can be sensed by the microglia which, in coordination with systemic inflammation, recruits inflammatory mediators to fight local impairments and triggers the release of a set of inflammatory mediators to install a pro-inflammatory phenotype in the long term. Microglia activation perpetuates a vicious circle for cytokine release, thereby installing a neuroinflammatory milieu. Created with BioRender.com, accessed on 27 September 2021.

Another relevant aspect of PD pathophysiology is related to peripheral inflammation and its possible link with central inflammation during neurodegeneration [160]. Being a first line of defense against microbial infections, the macrophage microglial cells sense cellular stress and damage and trigger cytokine production and ROS generation [161]. Mitochondrial-derived ROS have been indicated as major contributors to inflammation by the unloading of danger signals into the intra- or extracellular compartments in response to stressors [162,163,164]. These molecules, collectively indicated as damage-associated molecular patterns (DAMPs), are sensed via pattern recognition receptors (PRRs) and include, among others, the mitochondrial DNA (mtDNA). Increasing interest has been raised towards the analysis of circulating mtDNA as a DAMP molecule that activates innate immunity via the toll-like receptor (TLR) 9 at the levels of the endo-lysosomal membrane which triggers germ-free inflammation [165]. Mitochondrial-ROS mediated sterile inflammation may also be ignited via the activation of the NLR family pyrin domain, containing 3 (NLRP3) inflammasome and the stimulator of the interferon genes pathway [163]. This multidomain protein complex is formed by the procaspase-1 and the apoptosis-associated speck-like protein containing a CARD, referred to as ASC. Once activated, these two protein complexes cleave and release the active form of the proinflammatory cytokines interleukin (IL)-1β and IL-18. The NLRP3 inflammasome pathway activation may also induce the formation of pores in the plasma membrane that trigger a form of cell death called pyroptosis. In the long term, a sustained inflammasome activation may eventually lead to extensive tissue damage and the release of intra- and extracellular DAMPs that trigger inflammation. On their way back to the brain, the proinflammatory cytokines produced in the periphery cross the blood-brain barrier and enter the CNS, where they bind to the microglial receptors and stimulate the activation of NLRP3 and therefore induce neuroinflammation [166] (Figure 2).

According to this, neuroinflammation and mitochondrial dysfunction are tightly associated, and mitochondrial dysfunction may establish a vicious circle operating via ROS signaling. Indeed, dysfunctional organelles may instigate inflammation which, in turn, leads to further mitochondrial impairment and the consequent release of large quantities of mitochondrial DAMPs. The molecular mechanisms assisting in the mtDNA unloading are largely unknown; however, the mtDNA packaging into EVs and the EV delivery into the extracellular compartment as DAMPs have been described [164,167,168,169]. Along with mtDNA, other mitochondrial components have also been shown to compose the subset of extracellular DAMPs, including N-formyl peptides, cardiolipin, mitochondrial transcription factor A (TFAM), succinate, and adenosine triphosphate. Cell-free mtDNA and intact mitochondria holding functional competence have also been identified in the blood of healthy people [169]. 

Although mitochondrial dysfunction has been widely investigated in preclinical models of PD and signs of mitochondrial alterations have been reported in both sporadic and familial forms of PD, further studies are needed to evaluate the contribution of the release of mitochondrial DAMPs to systemic inflammation and to conclusively establish the role of this molecular pathway in PD pathophysiology [48]. 

## 7. Conclusions

The diagnosis of PD relies essentially on motor symptoms which, however, become detectable when 70–80% of the dopaminergic terminals in the striatum and about 50% of the dopaminergic neurons in the substantia nigra are lost. The existence of a long presymptomatic disease phase poses major obstacles to unveiling the molecular events responsible for the initiation and progression of neurodegeneration [46]. A wide range of clinical, biochemical, imaging, and genetic parameters have been investigated as possible biomarkers of PD [5,6]. Most studies were based on single marker approaches [7]. More recently, innovative multi-marker strategies have been applied to allow a more comprehensive appraisal of complex conditions, including PD [8,9,10,11,12].

Additional disease-specific biomarkers that could mirror the multifaceted characteristics of PD pathogenesis at the systemic level are highly sought after. To this end, ad hoc translational studies, including both pre-clinical and clinical information and the focusing on major PD-related mechanisms are needed. In particular, a deeper investigation of the signaling pathways involving α-syn, neuroinflammation, Aβ and Tau pathology, NfL, and axonal injury and degeneration may provide unprecedented information on PD pathophysiology and contribute substantially to the biomarker discovery process. A combination of α-syn species, lysosomal enzymes, markers of amyloid and Tau pathology, and NfL in the CSF and blood have been reported as potential diagnostic and prognostic biomarkers of PD, reflecting the dynamic course of its pathophysiology [4]. However, further validation of these mediators in large independent cohorts is required for their adoption in the clinical setting to improve PD diagnostic and prognostic accuracy. 

## Figures and Tables

**Table 1:** Summary of candidate biomarkers of Parkinson’s disease.

Biomarkers	Pathogenic Processes	Clinical Trial(s)	References
p-tau/total tau	Post-translational modifications of tau protein and p-tau pathology	−	[16]
p-tau/Aβ_42_	Post-translational modifications of tau protein, amyloid deposition and p-tau pathology	−	[16,17]
Aβ_42_/total tau, oligomeric α-synuclein/total α-synuclein	Amyloid deposition, α-synuclein, and tau pathology	−	[18,19]
Total tau/Aβ_42_	Amyloid deposition and p-tau pathology	−	[20]
NfL/Aβ_42_	Amyloid deposition, neurofilament light chain and axonal injury	−	[21]
NfL, Aβ_42_, p-tau, total tau, and total α-synuclein	Amyloid deposition, α-synuclein and p-tau pathology, and neurofilament light chain and axonal injury	PD01A (α-synuclein, NCT01568099), Nilotinib (α-synuclein and total tau, NCT02281474), MEDI1341 (total α-synuclein, NCT03272165), cerebral dopamine neurotrophic factor (α-synuclein different species, NCT03295786), Glycerol phenylbutyrate (α-synuclein, NCT02046434)	[22]
Oligomeric α-synuclein/total α-synuclein, phosphorylated α-synuclein and p-tau	Post-translational modifications (i.e., α-synuclein and tau phosphorylation)	KM-819 (oligomeric α-synuclein, total tau, p-tau, NCT03022799)	[23,24]
Total tau/total α-synuclein, p-tau/ α-synuclein, total tau/total α-synuclein with Aβ_42_, p-tau/total α-synuclein with Aβ_42_	Post-translational modifications (i.e., α-synuclein phosphorylation, amyloid deposition, and p-tau pathology)	−	[25]
DJ-1 with total tau and p-tau	Mitochondrial dysfunction and post-translational modifications	−	[26]
Glucocerebrosidase and β-hexosaminidase, cathepsin D, total α-synuclein, and Aβ_42_	Defective autophagy-lysosomal systems, and amyloid and alpha synuclein pathology	Ambroxol (GCase, NCT02941822)	[27]

Abbreviations: Aβ_42_, 42 amino acid proteolytic product from the amyloid precursor protein; GCase, glucocerebrosidase; NfL, neurofilament light; p-tau, phosphoprylated-tau.

## Data Availability

Not applicable.
